# Normal and Tumoral Melanocytes Exhibit q-Gaussian Random Search Patterns

**DOI:** 10.1371/journal.pone.0104253

**Published:** 2014-09-09

**Authors:** Priscila C. A. da Silva, Tiago V. Rosembach, Anésia A. Santos, Márcio S. Rocha, Marcelo L. Martins

**Affiliations:** 1 Departamento de Física, Universidade Federal de Viçosa, Viçosa, Minas Gerais, Brazil; 2 Departamento de Bioquímica e Biologia Molecular, Universidade Federal de Viçosa, Viçosa, Minas Gerais, Brazil; 3 National Institute of Science and Technology for Complex Systems, Rio de Janeiro, Rio de Janeiro, Brazil; University of Heidelberg Medical School, Germany

## Abstract

In multicellular organisms, cell motility is central in all morphogenetic processes, tissue maintenance, wound healing and immune surveillance. Hence, failures in its regulation potentiates numerous diseases. Here, cell migration assays on plastic 2D surfaces were performed using normal (Melan A) and tumoral (B16F10) murine melanocytes in random motility conditions. The trajectories of the centroids of the cell perimeters were tracked through time-lapse microscopy. The statistics of these trajectories was analyzed by building velocity and turn angle distributions, as well as velocity autocorrelations and the scaling of mean-squared displacements. We find that these cells exhibit a crossover from a normal to a super-diffusive motion without angular persistence at long time scales. Moreover, these melanocytes move with non-Gaussian velocity distributions. This major finding indicates that amongst those animal cells supposedly migrating through Lévy walks, some of them can instead perform q-Gaussian walks. Furthermore, our results reveal that B16F10 cells infected by mycoplasmas exhibit essentially the same diffusivity than their healthy counterparts. Finally, a q-Gaussian random walk model was proposed to account for these melanocytic migratory traits. Simulations based on this model correctly describe the crossover to super-diffusivity in the cell migration tracks.

## Introduction

Cell migration is a dynamic and complex process guided by a vast array of chemical and physical signals [1]. All nucleated cell types migrate at least during a given period of their development. In multicellular organisms, the regulation of cell motility is central in all morphogenetic processes, tissue maintenance, wound healing and immune surveillance [2]. Its failure potentiates numerous diseases, including inflammation, cardiovascular disease, cancer metastasis and various birth defects. Particularly, in metastatic solid cancers which are responsible for most disease mortalities, tissue cohesion is lost and both single and collective cell motility are enabled. Transformed, migrating cells rupture of basement membrane layers, invade adjacent tissues and migrate through interstitial matrices towards blood and lymph vessels [3]. On the other hand, creating artificial tissues and organs through the colonization of biomaterials by cells, requires the control of cellular organization, communication and movements [4]. So, to achieve the major goal of regenerative medicine it is imperative to characterize how cells move *in vivo* and understand the mechanisms that govern cell motile behavior.

Observations from a variety of cell types and experimental models reveal that cells move employing a continuum of migration modes, individual or collective. Mode selection is dictated by structural and molecular determinants of both tissue environment and cell response. Furthermore, motile cells can adapt and switch the modes of migration to distinct physiological and pathological contexts [5]. The switching can either be stochastic or elicited by extracellular factors, including therapeutic agents. The majority of these observations refers to single or densely organized cells moving on two-dimensional (2D) rigid surfaces *in vitro* [6]. Thus 2D cell motility assays provided us with most of our current understanding of cell migration. However, differences in cell motile behavior on 2D substrata versus within three-dimensional (3D) matrices were observed [7], [8]. The analysis of 3D cell migration is mainly based on fluorescence microscopy with optical sectioning capability. Nonetheless, this technique poses specific challenges related to cell labelling, fluorescence detection, and cell tracking [9]. Also, 3D cell motility assays have several limitations concerning the systematic comparison of the major 3D matrix models, control of their physicochemical features, resolution in the z-axis imaging, and standardization of quantification routines [10]. In particular, the automatic recognition of distinct migratory phenotypes is a challenge for functional genomic screening approaches [11] to cell migration.

From the physicists stand point, individual cell migration can be mapped on a search process for targets (e. g., nutrients, growth factors and chemokines), detectable by the cell only within a limited spatial range [12]. In the absence of external gradients of such cues, motile cells perform random walks whose characteristic features probably reflect some of the molecular and subcellular mechanisms that regulate their migration phenotype. Hence, the systematic analysis of experimental time series for trajectories of migrating cells will yield much quantitative information for generate cell-type specific motility models. These “macroscopic” models of cellular behavior integrated with “microscopic” descriptions of the dynamics of adhesion molecules, cytoskeleton remodeling and generation of traction forces [13] will constitute the systems biology of cell motility.

In this context, the movement of several cell types, from unicellular to multicellular organisms, were characterized. It has been found that cells commonly migrate with a directional persistence generating correlated random walk patterns [14]. This is the case, for instance, of *Dictyostelium* [15], [16], Hydra [17], human mammary epithelial cells [18], fibroblasts and keratinocytes [19]. In contrast to the Ornstein-Uhlenbeck process [21], maybe the simplest and most popular model for persistent random walks, some of these cells [15], [19], [20] exhibit non-Gaussian velocity distributions. Furthermore, micro-organisms and cells of the immune system can perform Lévy walks, a special case of superdiffusion in which the distribution of step lengths has infinite variance [22]. So, for example, the dinoflagellate *Oxyrrhis marina* executes Lévy flights when its prey decreases in abundance [23]. Also, the movement of 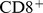 T cells in the brains of mice infected by *Toxoplasma gondii* is well described by an intermittent Lévy walk [24]. However, T and B cells migrate within intact lymph nodes by a normal random walk [25]. Summarizing, the motion of cells is rich in variety and no single universal search strategy applies to all cell types and environmental conditions.

In the present paper, we performed cell migration assays on plastic 2D surfaces using normal and tumoral murine melanocytes plated at low densities. Experimental time series for individual trajectories of migrating cells were recorded by time-lapse microscopy. From these trajectories, velocity and turn angle distributions as well as velocity autocorrelation functions were determined. Our major finding is that murine melanocytes perform q-Gaussian walks. This result raises the possibility that some cells, previously considered as Lévy wanderers, can instead migrate through q-Gaussian walks. Additionally, we investigated the effects of mycoplasma contamination on the motility of B16F10 melanocytes. Our motivation was the observation made by Murooka *et al*. that HIV induces a reduction in the motility of infected T cells [26]. By tuning the migratory and interactive behavior of T lymphocytes, the HIV viruses enforce T cells to serve as vehicles for efficient local and systemic viral dissemination. Concerning mycoplasma infection, the consequences for the host cells vary widely, from no apparent effect to inhibition of metabolism and growth up to induction of apoptosis or malignant transformation [27], [28]. But mycoplasma impair the motility of infected cells? Our main result is that the murine melanocytic migration mode is not affected by mycoplasma infection. Maybe, similar studies can provide valuable insights about the use of viral or bacterial agents to impair cancer cell motility and invasion or identify molecular targets against metastatic cells.

## Materials and Methods

### Cell culture

Melan A cells, a murine immortalized melanocyte line (São Paulo State Cancer Institute, São Paulo, SP, Brazil), B16F10 cells, derived from a murine melanoma (Pharmacology Department, Minas Gerais Federal University, Belo Horizonte, MG, Brazil), and B16F10 cells contaminated by mycoplasma were used. These cells were cultured in 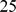 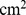, 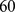 ml flasks (Techno Plastic Products AG 90025) at 37°C with 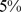 of 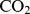 in Dulbecc's Minimum Essential Medium (Sigma Aldrich) supplemented with 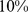 fetal calf serum (Cultilab, Campinas, SP, Brazil), 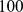 i.u./ml penicilin, 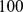 *µg*/ml streptomycin, 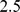 ng/ml amphoterycin B, 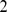 mM glutamine, 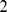 mM sodium pyruvate, and 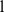 mM non-essential amino acids. For Melan A cells, 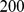 nM 12-o-tetradecanoyl forbol 13-acetate (PMA, Sigma-Aldrich) was added.

Cells, with a typical length (major axis) of 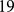 *µm*, were sparsely seeded at a small number (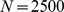 cells) on the plastic surface of the flasks, corresponding to a very low density of 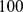 cells per 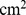. All migration assays had 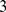 to 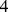 biological replicates and were performed without any externally established chemo-attractant gradients.

### Time lapse microscopy

Cell displacements were tracked via an inverted Nikon TS 100 phase-contrast microscope equipped with a CCD camera (JAI CM 140 GE) with an electronic magnification of approximately 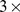 and a 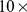 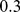 NA air objective. Data were collected at a resolution of 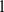 pixel 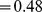 *µm* from a fixed imaged field with 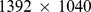 pixels. Imaging started 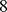 hours after cell plating with a video-microscopy sampling interval of 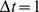
min and typically last for 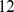 h. Only cells that did not adhere to other cells, or undergone division or death or moved out of the imaged field were included in the analysis. As a result, the number of cells filtered for tracking procedure was 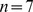 for Melan A, 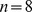 for B16F10 and 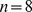 for contaminated B16F10 cells. Supposedly, these tracked cells have intermediate migratory capacities. Indeed, neither the lower nor the higher motile cells are sampled by the criteria considered for tracking procedure. However, accordingly our qualitative observations, the migratory cells visualized in our assays exhibit rather homogeneous behaviors.

For each cell trajectory 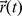, the positions 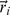 of the cell contour centroid at the times 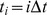 (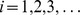) were recorded. The corresponding velocities were calculated as 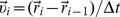. From these data, velocity distributions and autocorrelation functions, as well as the probability distributions of the turn angles within cell trajectories were determined.

### Data analysis

The speed distribution 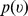 was defined as the fraction of velocity data points with speed 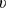 binned between 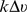 and 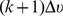. The value of 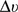 was chosen as described below and 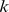 was varied from 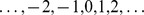. In order to fix the same average and standard deviations for the speed distributions exhibited by distinct cells within a cell line, instead of 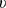, 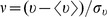 was used. Here 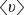 and 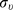 are the speed average and its standard deviation for each cell tracked, respectively. Then, the distribution 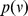 was built as a histogram with a fixed bin size 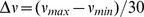. The number 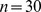 was empirically chosen aiming to generate the largest number of bins, each one containing statistically significant sample of data points.

The velocity autocorrelation function was defined as



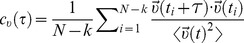
(1)where 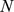 is the total number of data points and 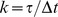 is the number of sampling intervals associated to the time lapse 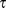 considered. As in reference [15], 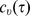 was calculated for each trajectory as



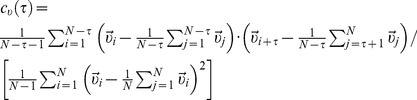
(2)in order to minimize the effects of noise on time-lapse recorded positions which otherwise leads to a negative 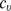 at small 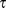 values.

The instantaneous turn angle 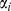 was defined as follows. If 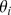 is the orientation of the displacement 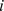, 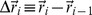, relative to the horizontal axis, then 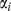 is given by 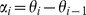. So, the turn angle distribution 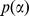 was defined as the fraction of trajectory steps for which their instantaneous directions changed by an angle 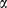 between 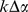 and 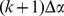 relative to their previous steps (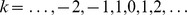). A histogram for the turn angles with a bin size 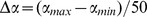 were built. Again, 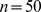 was empirically chosen to generate a large number of bins, each one containing a significant sample of data points.

As done in reference [18], we analyzed if the cellular migration exhibits alternating modes (directional and re-orientation phases). Here, we adopted the following mode definition. From the turn angles 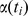 of an individual cell trajectory and a chosen threshold value 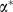, a directional “flight” starts at a time point 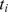 if at least 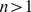 successive steps have 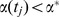, 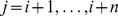. In turn, a re-orientation flight begins at 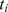 if 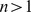 successive time steps have 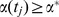. The values *α* ^*^ = 5°, 15°, 30°, 45°, and 60° were chosen. Once the migration modes have been defined, their distributions of flight lengths were determined as the fraction of flights with contour lengths 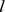. The contour length 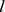 of a flight starting at a time point 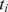 is defined as the total distance it traverses. Therefore, 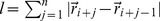, where 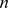 is the number of steps comprising the flight.

### Statistical analysis and simulations

A multivariate analysis based on Hotelling 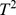 test [29] was used to compare the vector of means (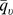, 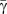) for the indexes 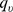 and 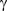 (see the next section) characterizing each cell lines considered. In addition, experimental cell tracks, displacement and turn angle distributions, and mean-squared displacements were qualitatively compared with those data obtained from computer simulations of q-Gaussian walks. In such walks, a first randomly oriented step is followed by stochastic sequences of directional flights punctuated by single re-orientation jumps. These re-orientation steps, performed with a probability 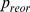, have their directions randomly chosen independently from those of the previous steps. In turn, a directional flight, performed with a probability 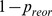, is comprised of 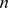 successive correlated steps. The value of 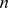 is drawn from an exponential distribution 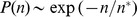. Each step in a flight randomly deviates from the orientation of the last step performed before the flight starting by at most 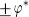. Two senses of direction are possible for the flight: either anti-parallel (reversion), chosen with a probability 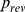, or parallel (persistence), chosen with a probability 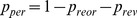, to the direction of the last step performed before the flight starting. Finally, every step has a length randomly chosen from a q-Gaussian distribution [30].

## Results

**Figure 1:**
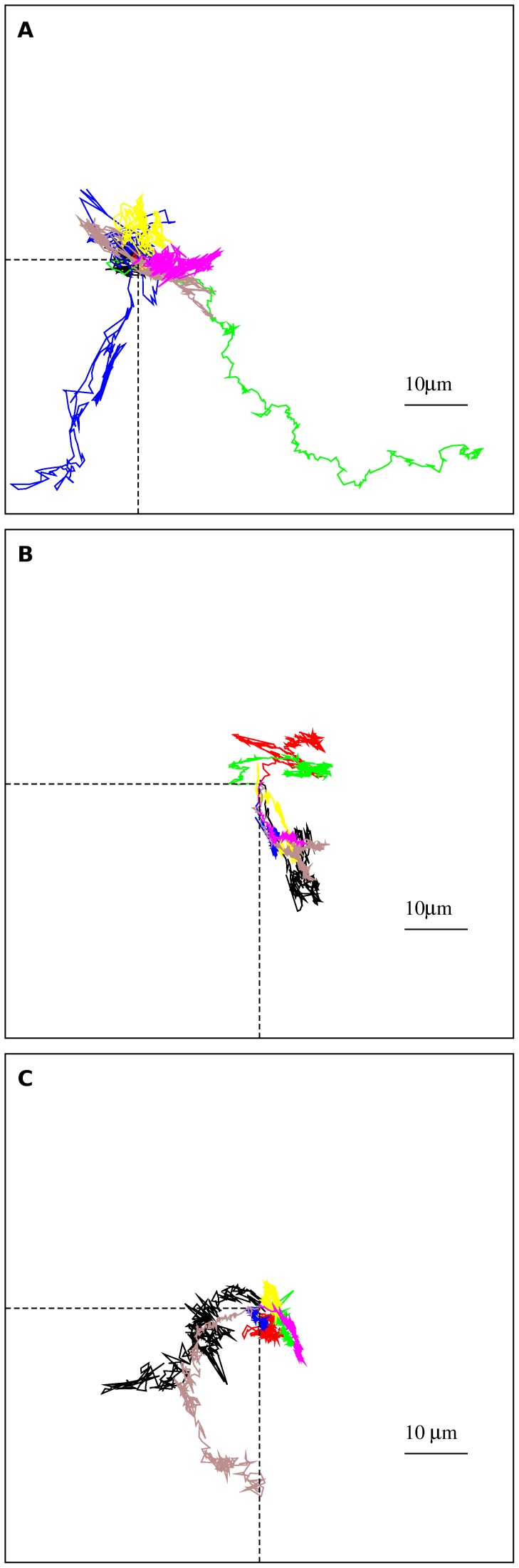
Typical migration tracks on 2D plastic substrates. (a) Melan A (normal), (b) B16F10 (tumoral), and (c) contaminated B16F10 cells. The trajectories were produced by time-lapse recording of cells every 1 min and plotted from the origin. The error 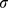 due to pixel round-off errors on our estimates of the centroid positions is 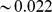 *µm*, corresponding to about 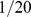 of a pixel width (see Text S1, Figure S1 and Figure S2).

**Figure 2:**
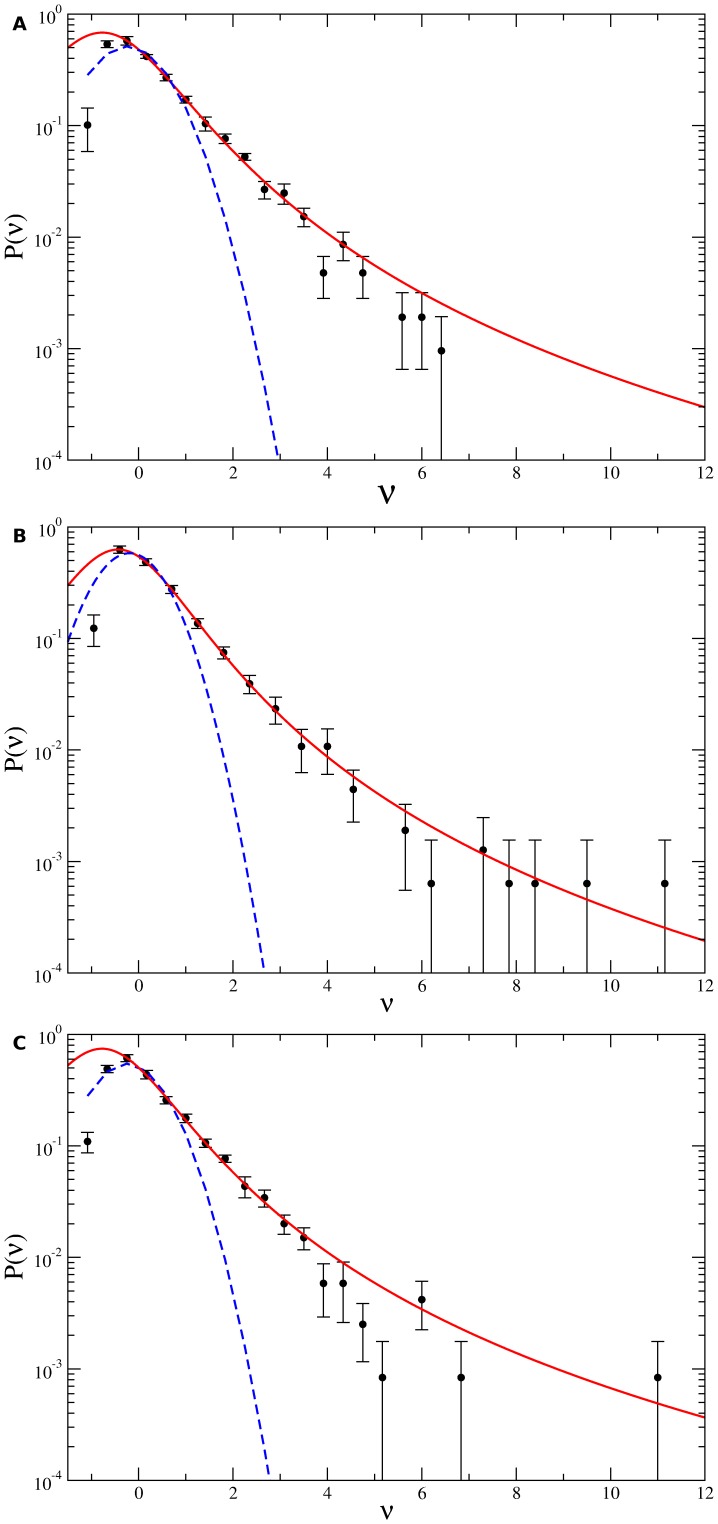
Experimentally measured ensemble speed distributions. (a) Melan A, (b) B16F10, and (c) contaminated B16F10 cells. The distributions were plotted with log-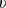 axis and the solid curves are q-Gaussian fits to data. For comparison, Gaussian distributions fitted to data are shown (dashed curves). The velocities for every individual cell of a given type were merged to form single large data sets.



In Figure 1 typical trajectories for each of the cell lines tested are shown. The positional errors associated to these trajectories were discussed and estimated in the Supporting Information (see Text S1, Figure S1 and Figure S2). We found that Melan A, B16F10, and contaminated B16F10 cells migrate at average speeds of 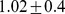 *µm*/min, 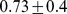 *µm*/min, and 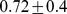 *µm*/min, respectively. The speed distributions are depicted in Figure 2. The random migration of normal and tumoral melanocytic cells **are** characterized by q-Gaussian distributions [31] expressed by the formula

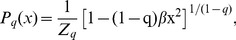
(3)with 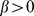 and



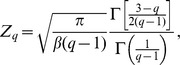
where 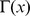 is the Gamma function. The q-values characterizing the speed distributions are listed in Table 1.

**Table 1:** q-indexes, exponents and fitting parameters to cell diffusion data.

Cell type	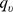	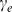	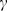	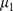	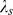	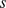
Melan A	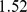	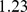	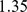	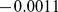	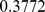	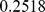
B16F10	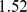	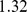	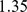	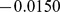	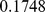	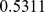
B16F10 contaminated with mycoplasma	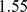	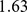	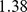	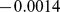	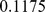	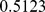

Ensemble 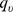 values and 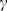 exponents characterizing the q-Gaussian cell speed distributions and the scaling in time of the mean-squared cell's displacements. The exponents 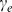 are the slopes of the empirical power-law fittings to average 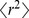 curves. The 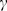 values were predicted from the speed distributions through the expression 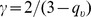. Also, the parameters 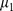, 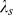, and 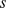 used for fitting the experimental data for average 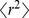
's are listed.

**Figure 3:**
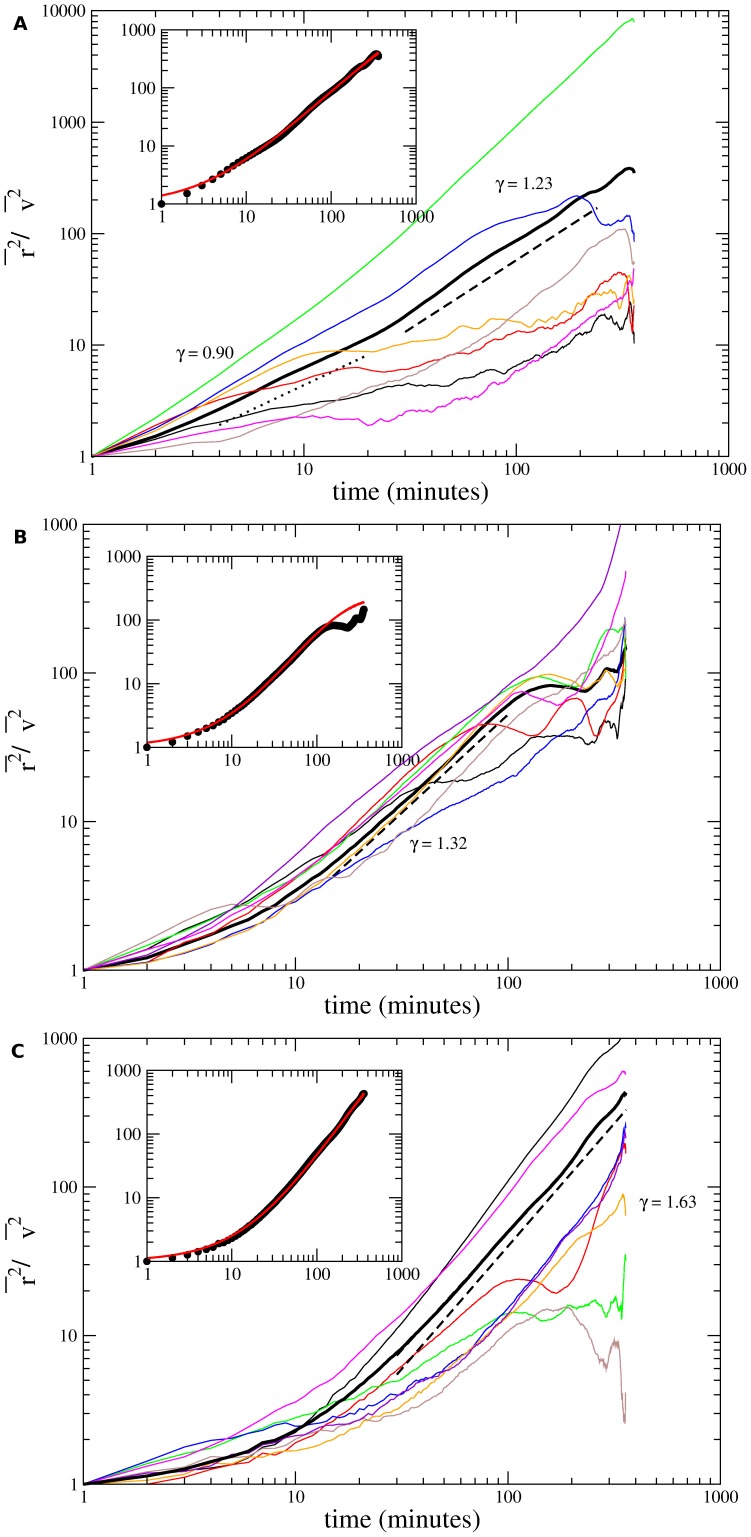
Experimentally measured mean-squared displacements 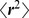 as functions of time. (a) Melan A, (b) B16F10, and (c) contaminated B16F10 cells plotted with log-log axis. The colour curves correspond to distinct cell trajectories. The thick black curve is the average of all trajectories. The dashed lines are power law fits to data whose slopes provide the exponents 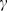 characterizing the migratory regimes. Mean-squared displacements were divided by 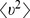 in order to put in the same scale cells with very distinct motilities. Insets: Average mean-squared displacements fitted by Equation 5. A crossover from a normal to a supperdifusive regime is indicated.



The anomalous (non-Brownian) character of these migrations is confirmed by the scaling in time of the mean-squared displacement given by



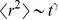
(4)with 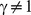. In Figure 3 are shown log-log plots of mean-squared displacements for distinct individual cells of the three cell lines as functions of time. Power-law fittings to empirically chosen linear portions of the average 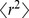 curves were drawn (dashed lines) and their slopes 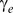 were indicated. In the insets, we have fitted the whole set of experimental data for the average mean-squared displacements over all cells within a cell line. The fits assume a crossover between two different power-laws [32]. Specifically, if 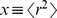 satisfy the equation

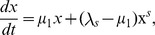




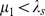
, its solution



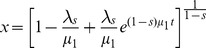
(5)provides the parameters 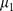, 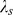, and 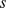 for fitting the experimental data. The red curves shown in the insets correspond to such fits and their parameter values are listed in Table 1.

**Figure 4:**
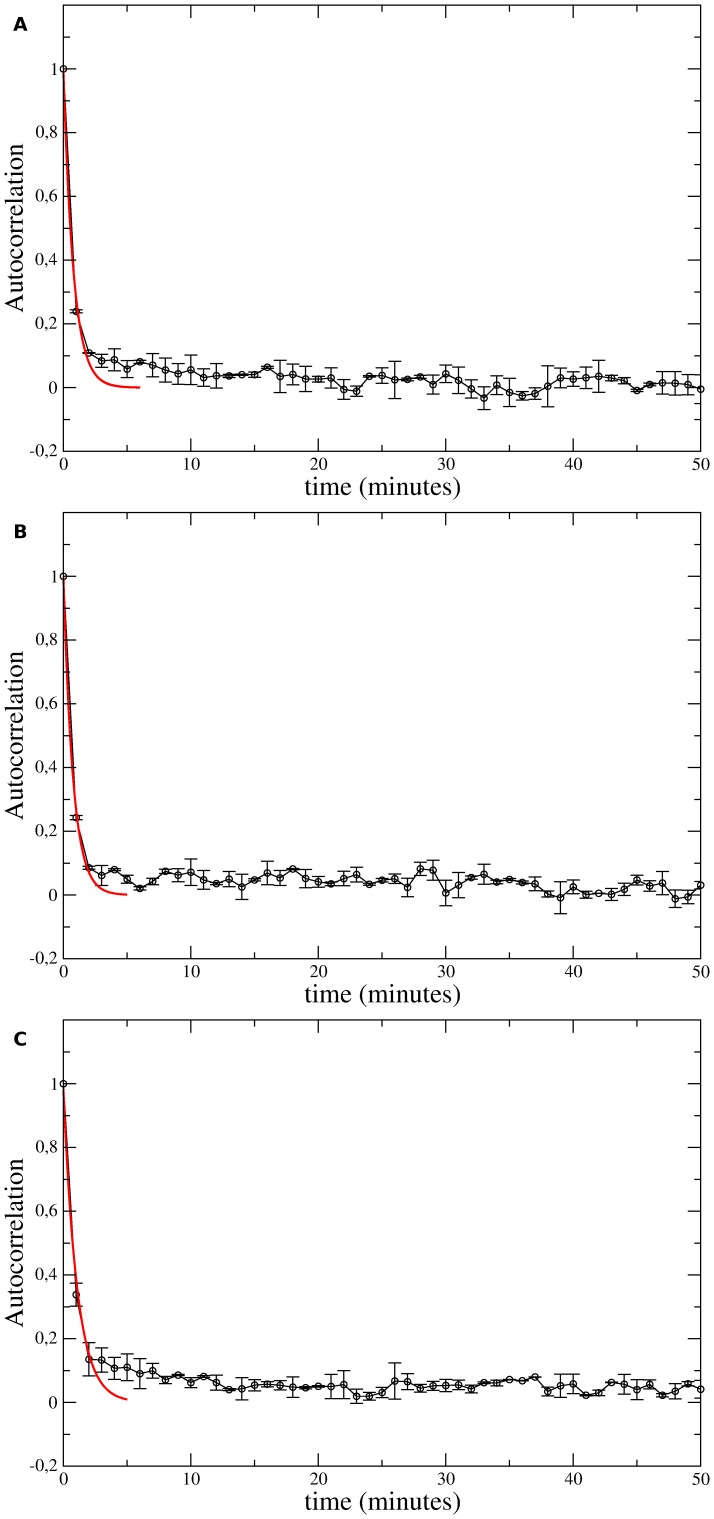
Typical velocity autocorrelation functions. (a) Melan A, (b) B16F10, and (c) contaminated B16F10 cells. Velocities were defined as (displacement vector)/(time-lapse) for each 1 min time-lapse of each cell's trajectory. The red curves correspond to exponential fittings to the data at short-time regions.

In order to check the presence of correlations in the migration patterns of these cells, we also measured the velocity autocorrelation function through eq. (2) and the probability distributions of the turn angles between successive time steps. Figure 4 shows our experimental results for the velocity autocorrelation functions. The cell lines tested exhibit correlations decaying exponentially in time with very short characteristic time scales, typically 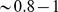
min.

**Figure 5:**
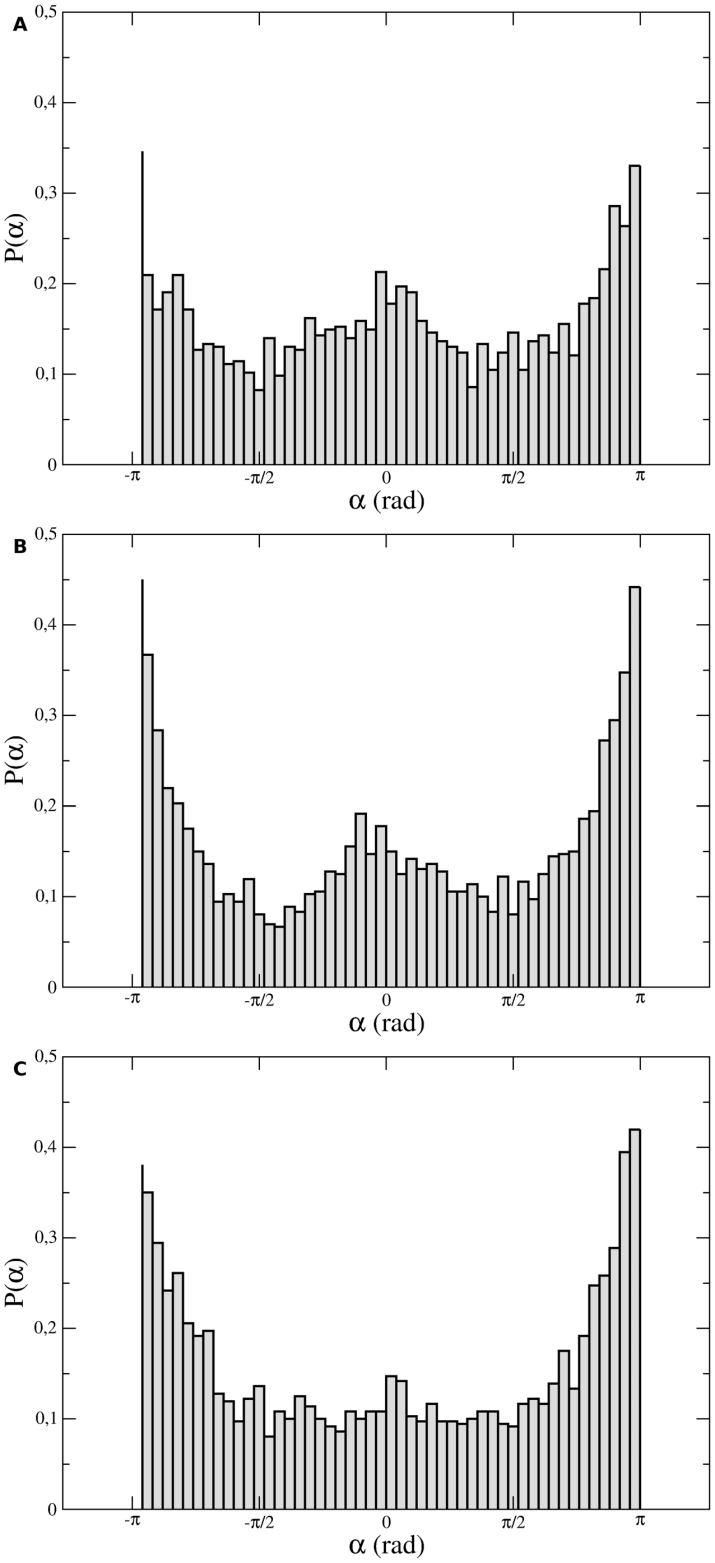
Turn angle distributions. (a) Melan A, (b) B16F10, and (c) contaminated B16F10 cells. The instantaneous turn angle was defined as the difference between successive displacement orientations for each 1 min time-lapse of each cell's trajectory.

Furthermore, the turn angle distributions, illustrated in Figure 5, are “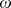-shaped” curves with local maximums at small (around 0°) and large angles (around ±180°). Despite these preferences for small and large turns, it must be observed that intermediate turn angles have significant probabilities. The shapes of the turn angle distributions suggest a bimodal analysis in which every individual cell track is subdivided into directional and reorientation “flights”. The former (latter) are comprised of successive displacements whose turn angles are always smaller (greater) than a fixed threshold [18]. Our results reveal that both flight types are exponentially distributed for turn angle thresholds 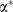 ranging from 5°–60°, supporting the robustness of these findings (see Text S1, Figure S3, Figure S4, Figure S5 and Figure S6). The directional flights have a small characteristic number of steps and, consequently, short characteristic contour lengths 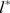. For instance, Melan A cells exhibit 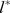 varying from 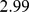 *µm* for *α* ^*^ = 15° to 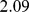 *µm* for α^*^ = 60°. Furthermore, turn angle distributions for reorientation flights have maxima at ±180° and the widths of directional flights increases as the threshold 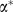 increases (see supporting information). It was found that an increase in 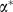 increases (decreases) the number of directional (reorientation) flights and their average traversed distances. However, for both flight types their characteristic contour lengths decrease. For instance, 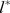
's decreases from 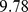 *µm* for 15° to 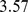 *µm* for 60° in reorientation flights of Melan A cells.

**Figure 6:**
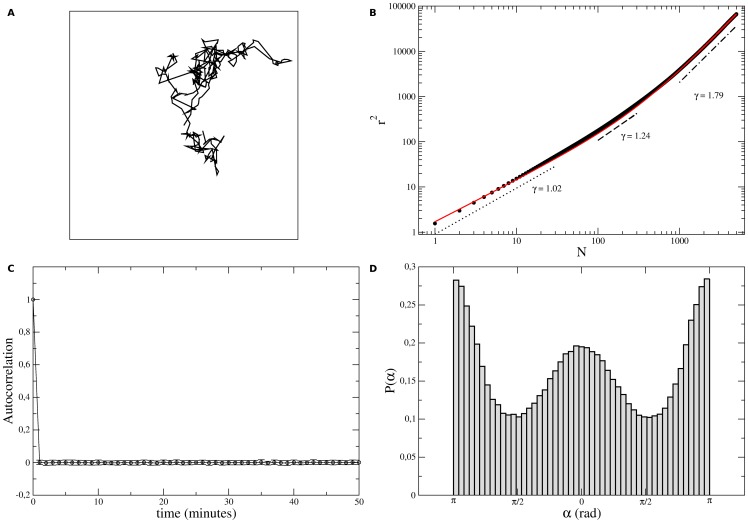
Simulation results. (a) Typical track, (b) mean-squared displacements as functions of time, (c) velocity autocorrelations functions, and (d) turn angle distributions obtained from the simulations of cell walks. The parameter values used were 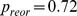, 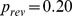, 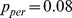, 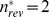, 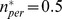, and *φ* ^*^ = 30°. For comparison with cell tracks, the simulated walk in (a) contain around 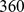 steps, but 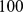 independent samples with 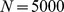 steps were averaged to determine the statistical properties of the simulated walks. In (b), the red curve is a fitting to the data given by 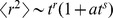. We find 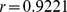 and 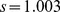, thus corresponding to a crossover between a Brownian and a ballistic walk.

Finally, the results of our simulations are shown in Figure 6. As can be noticed, the simulated tracks, turn angle distributions, and velocity autocorrelation functions are qualitatively consistent with the experimental results. In particular, the velocity autocorrelation is a delta function in agreement with the very short correlations in cell's velocities experimentally observed. Also, the correct shapes of cell turn angle distributions demand the occurrence of short persistent or anti-persistent directional flights in the simulated walks. These flights asymptotically lead to ballistic trajectories characterized by mean-squared displacements scaling as 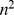 for large number of steps 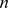. Nevertheless, as observed for the cells tested, at intermediate time scales the mean-squared displacement scales as 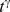 with 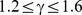 for 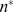 ranging from 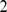 to 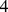. This variation in time of the exponent 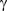 is, indeed, the result of a crossover between normal and ballistic diffusion regimes, as the fitting of the simulational results for 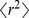 demonstrates (see Figure 6 (b)). Thus, supported by our simulations, we hypothesize that this crossover, neatly observed in the experimental data for B16F10 cells, also occurs in Melan A cells, but slowly.

## Discussion

We examined the migration (or search strategies) of normal and tumoral murine melanocytes plated on plastic 2D surface free from any external signaling gradients (random motility conditions). Indeed, cells were plated at very low density so that the average distances between them are more than 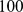 cell diameters. Our analysis was focused on the scaling of the cell's mean-squared displacements and the statistics of cell's speed and turn angle distributions. Under random motility conditions, these epithelial cell lines demonstrate a rather constrained translational migration superimposed with significant cell shape fluctuations, as can be seen in Movie S1. Currently, we are performing a quantitative analysis of such cell shape fluctuations. Whatever is the relative strength of these contributions, we found that the centroids of the cells exhibit a super-diffusive motion without angular persistence at long time scales and with non-Gaussian speed distributions. Also, our statistical analysis showed that there is no significant difference at any level of significance equal or greater than 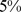 between Melan A, contaminated and non-contaminated B16F10 cells.

Concerning cell's velocities, apart from very short initial anticorrelations they are uncorrelated at long-term and distributed as q-Gaussians. Their 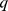 indexes are in the range 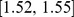 indicating, because 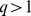, super-diffusion. Thus, the normal and tumoral murine melanocytic cell lines tested perform superdiffusion (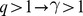). Furthermore, our results demonstrate that there is place for q-Gaussian anomalous diffusion of the kind reported on reference [20]. We hypothesize that the ubiquity of persistent random walks in cell migration is a consequence of cell density. If such densities are not too small, each cell is subjected to chemotactic gradients generated by other cells not so far away. This cell-cell signaling can lead to correlated random walk patterns as observed in reference [18]. In this study, cells were plated at a density of approximately 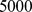 cells per 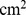, fifty times the density used in our experiment. However, at very low densities, normal diffusion can dominate at the longest time scales as found in reference [15]. In this experiment, the initial average distances between individual cells are more than 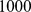 cell diameters. In the literature anomalous cell diffusion, primarily associated to correlated random walks, is also often attributed to Lévy motion wherein long steps (flights) are intercalated by shorter jumps. These walks are characterized by power law distributions for the displacements [24]. Strictly speaking, this is abusive because a power law tail does not imply a Lévy distribution. Indeed, if 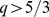 where it is possible to compare q-Gaussian and Lévy distributions, both exhibit asymptotic power law decays given by 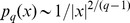 and 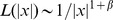, respectively. Even worse, if the indexes 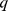 and 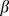 are related through 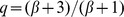 the tail of both distributions decay with the same power-law exponent [31]. Hence, for 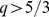, it may become experimentally very difficult to distinguish between these two distributions. Thus, it is possible that amongst those animal cells supposedly migrating through Lévy walks, some can instead perform q-Gaussian walks.

Regarding the mean-squared displacement as a function of time, the theoretical expression 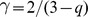, relating the mean-squared displacement exponent 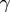 and the 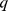 index [31], leads to exponents 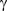 in the range 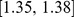. Such values are in good agreement with those provided by the empirical power-law fittings at intermediate times (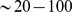
min) for Melan A and B16F10 cells. However, for contaminated B16F10 cells, the theoretical value deviates significantly from the corresponding power-law fit to the data. Moreover, as suggested by the insets of Figure 3 and the fits to short- and intermediate-time regions for Melan A, a crossover between two different power-laws is consistent with the experimental data. Hence, we have fitted the whole set of data by using equation 5. This equation exhibits short- and intermediate-time regions whose asymptotic behaviors respectively are 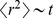 and 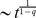. Thus, the short-time regime corresponds to a normal diffusion (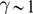), whereas in the intermediate-time scale the diffusion is anomalous (non-Brownian). Specifically, it is ballistic (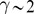) for B16F10 cells, contaminated or not, and superdiffusive (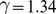) for Melan A cells. Supported by our simulations, we hypothesize that Melan A cells will also reach a ballistic regime, but slowly.

This crossover occurs around 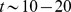 min and can also be observed in our simulations of q-Gaussian walks at the same scale. It seems that this crossover simply reflects the fact that for small displacements a q-Gaussian is almost identical to a Gaussian. So, apparently there is no biological mechanism related to cell motility acting as the source of this crossover. However, it is tempting to attribute such crossover to chemotaxy. Accordingly, the plated cells should initially perform random searches until they start to detect signalling gradients progressively established in the culture plate. Once chemotactic gradients have been established, the cells in response turn to a directed migration mode. At long-term, our simulations generate an asymptotic ballistic regime in which 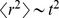 providing additional support to our experimental findings. Such long time scales are reached in our experiments, and this asymptotic behavior is consistent with the turn angle distributions observed for the cells tested. Indeed, the turn angle analysis reveal that Melan A and B16F10 cell lines exhibits a slight preference for either persistent (directional) motion or reversions (tumbles). This preference generates local maxima in the turn angle distribution centered around 0° and ±180°. However, intermediate values of the turn angle are also very probable, leading to a significant, almost uniform distribution inbetween these three peaks. In addition, the sequences of persistent jumps are very short with displacements exponentially distributed and small characteristic lengths. In comparison with tumbles and reorientation sequences, persistent flights are rare (see Text S1).

The directional flights, responsible for either revertive or progressive motions, shape the experimentally observed turn angle distributions 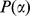 as the simulations demonstrate. Furthermore, the characteristic lengths 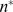 of these directional flights determine the heights of 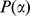 central peaks. The larger is 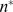, higher is the peak of 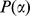 centered in 0°. Also, the simulations confirm that persistent flights lead asymptotically to a ballistic migration. These persistent flights can be associated to large cell elongations forward and backward along directions that change slowly and randomly. The Movie S1 attached to the supporting information illustrates the dynamics of cell shape alteration and pseudopod protrusion under random motility conditions. It seems that under very weak chemotactic gradients, long-term cell polarization and stable focal contacts are not established, consequently impairing migratory persistence.

For contaminated B16F10 cells, we find that the persistent flights are absent and, in consequence, the peak centered around 0° in the corresponding turn angle distribution vanishes. In addition, the average empirical exponent 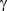 for contaminated B16F10 cells is larger than that characterizing the mean-squared displacement of healthy B16F10 cells. This indicates that contaminated B16F10 cells reach the ballistic regime slightly faster than their normal counterparts. So, their effective diffusivity remains unaffected, just the opposite effect of HIV virus on infected T cells. Finally, mycoplasma contamination leads to decreasing frequencies of highest velocities in comparison with those predicted by the associated q-Gaussians. It is known that the interaction of mycoplasma with target cells trigger cytoskeletal rearrangement in these host cells [33]. The belief is that this reorganization of the eukaryotic cell cytoskeleton promotes mycoplasma internalization. Our findings suggest that this reorganization can also enhances cell motility by suppressing persistent flights which, for B16F10 cells, mainly include revertive movements. This scenario is confirmed by our simulations (see Text S1, Figure S7 and Figure S8). Further, this mechanism overcomes the smaller average speeds of contaminated cells due to lighter tails exhibited by their velocity distributions.

### Biological implications of our findings

In this subsection we will situate our results obtained from the analysis of melanocyte migration within the conceptual scenario for cell motility.

Previous studies, mainly focused on highly motile cells (lymphocytes, fibroblasts, keratinocytes, and amoebae), revealed essentially two individual migration modes: correlated random walks (CRW) [14] and Lévy walks [35]. Once external directional signals (chemotactic or mechanical gradients) have been sensed, motile cells adopt biased CRW. Such walks exhibit long-ranged directional persistence that produces quasi-ballistic trajectories. These straight-line movements with variable low amplitude random behavior minimize the average distance travelled by the cells before first encountering the targets. Hence, highly efficient cellular mechanisms for sensing chemical gradients were selected through evolution.

However, before cells sense a graded signal or in the absence of such cues, in what individual migration mode they engage? In addition to their own characteristics, the performance of a search algorithm depends on extrinsic factors such as the dimensionality and spatiotemporal scales of the search landscape, density and dispersion pattern of the targets, and encounter dynamics. Lévy search seems to be the most efficient strategy to find revisitable targets, i. e., targets that stand for future searches [36], [37]. Accordingly, effector lymphocytes, recruited to the site of an inflammation, chase for pathogens by performing Lévy walks [24].

Nevertheless, in two and three dimensions, Lévy walks and ballistic movements can be equally effective for imperfect destructive searches of randomly and sparsely distributed targets [38]. A search is destructive when the targets are not revisited due to either depletion or rejection, and it is imperfect when there are target detection errors. Further, Lévy walks outperform ballistic motions in imperfect destructive searches when targets can occasionally evade capture once detected [38]. So, based on these theoretical evidences, it should be expected that in environments with low cell densities and free from external chemotactic gradients (random motility conditions), cells will perform either ballistic or Lévy searches.

The experiments on cell migration under random motility conditions indicate the following.

**Figure 7:**
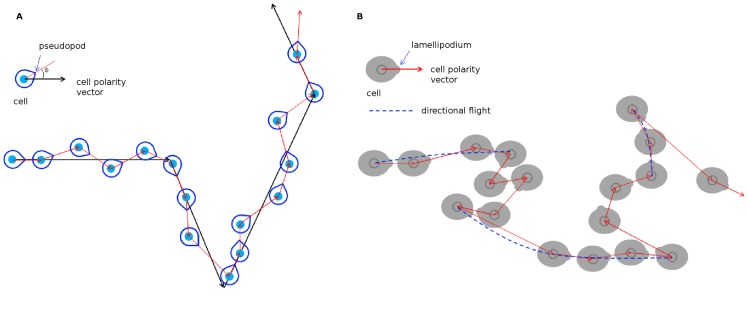
Models for cell migration. (a) The CRW model for Dictyostelium migration [16]. Successive pseudopods are protruded in a left-right-left-right fashion around the cell's polarity direction, propelling the cell forward in zig-zag. In turn, the direction of cell polarization changes randomly (with uniform distribution), but slowly over time. (b) The CRW model for melanocyte motion. The cell's polarity changes randomly, but with preferred directions, either 0° or 180°, generating progressive and revertive runs. Also, because the cell speed distribution is a q-Gaussian, the histogram of step lengths is heavy-tailed. So, melanocyte exploited stochastic factors related to both displacement lengths and turn angle distributions to enhance their search capabilities.

- The amoeba *Dictyostelium discoideum* and *Polysphondylium pallidum* perform CRWs with persistent times 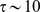 min and non-Gaussian (Cauchy) speed distributions [15], [16], [39]. Specifically, these amoebae exhibit a crossover from an oscillatory random motion at short times to a purely diffusive Brownian motion on the longest time scales (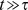). They move forwarded in a zig-zag manner fluctuating around the cell polarity directions that turn every 1–2 min away from the last polarization vectors: a left turn tends to be followed by a right turn. Combined with persistence, this turn-run-turn migration results in a fairly straight motion of the amoebae, significantly improving their search efficiencies. See Figure 7 (a). Indeed, Dyctyostelium cells are approximately half as efficient as ballistic search, and 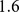 to 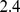 fold more efficient than random walk searching [16].
- Transformed human keratinocytes (HaCaT cells) and normal human dermal fibroblasts (NHDF cells) perform CRWs with non-Gaussian velocity distributions and accelerations that, instead to be uncorrelated Gaussian noises, exhibit small correlations own to the memory of past velocities [19]. This memory relies on the direction of a moving cell encoded in the polarity of its cytoskeleton, and it lasts longer than individual transient pseudopods. It was suggested that such cell motions can be described by a model that realizes Lévy walks as Markovian stochastic processes [39]. Thus, since Lévy walks are optimal when searching in the absence of external cues and without prior knowledge of target locations, these movements should be strongly selected by evolution.
- Our findings for transformed (Melan A) and tumoral (B16F10) melanocytes indicate that these low motile epithelial cells migrate through CRWs with broad turn angle distributions peaked in 0° and 180°, therefore favoring progressive and revertive steps. In consequence, melanocytes move via random sequences of exponentially distributed re-orientation and directional runs. The persistence time in directional (revertive/progressive) runs are short. The speeds of the cells at search steps follow q-Gaussian distributions that have lower decay rates in comparison to a Gaussian (“fat” tail). The overall result is a crossover from normal diffusion at short time scales to a ballistic motion at long times. For B16F10 cells, revertive steps are less frequent and they migrate faster than Melan A cells. This migratory advantage may be relevant for cancer progression. Also, such a crossover has a biologically appealing insight as ballistic walks can optimize random searches. Perhaps, this is the case with epithelial cells that quest for and adhere to one another in order to form a new tissue. Similarly to the amoebae, our observations indicate that melanocytes do not sustain stable cell polarity directions. They protrude lamellipodia in random directions. These structures lives for short stochastic periods and promote persistent displacements of the cell's centroids around their axes. After the retraction of a lamellipodium, another one is protruded in a new direction randomly chosen, but preferentially either parallel or opposite to the previous polarity axis. A cartoon describing the melanocyte motion is shown in Figure 7 (b). Its worth to notice that these melanocyte's broad histograms of turn angles support the simulational results obtained by Bartumeus *et al.* [40], according to which excessively sharp distributions generate inefficient searches in landscapes where the target densities fluctuate largely in space.

In summary, the trajectories of normal and tumoral murine melanocytic cells were tracked through time-lapse microscopy. The speed and turn angle distributions associated to such trajectories, as well as their velocity autocorrelations and the scaling of their mean-squared displacements were analyzed. We find that these cells exhibit a super-diffusive motion with non-Gaussian velocity distributions and without angular persistence at long time scales. In addition, we find that B16F10 cells infected by mycoplasmas exhibits essentially the same diffusivity than their healthy counterparts. A q-Gaussian random walk model was proposed to account for these cell search patterns. Its simulation provided results that correctly describes the super-diffusivity in the cell migration tracks. From an evolutionary stand point, our results give support to the hypothesis that life organisms, from individual cells to animals, employ correlated random motions in order to maximize their success in non-oriented searches. In particular, melanocytes have adapted their movements exploiting displacement lengths and turn angle distributions to approach ballistic trajectories under random motility conditions.

## Supporting Information

Figure S1
**Positional error estimate for B16F10 cells using Li** ***et al*** **aliasing correction.** Average spectrum for B16F10 cells (black curve). It was fitted with the aliased 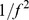 spectrum plus the positional noise spectrum (blue curve). The power-law fitting 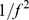 is the dashed red line and the aliased 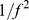 behavior is the red curve.(TIFF)

Figure S2
**Positional error estimate for B16F10 cells using Kirchner aliasing correction.** Average spectrum for B16F10 cells (black curve). It was fitted with the aliased 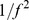 spectrum suggested by Kirchner [34] plus the positional noise spectrum (blue curve). The power-law fitting 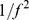 is the dashed red line and the aliased 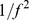 behavior is the red curve.(TIFF)

Figure S3
**Flight length distributions for Melan A cells.** Probability of flights having lengths greater than 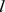 as function of 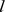 for Melan A cells. The turn angle thresholds used were (a) *α* ^*^ = 15°, (b) *α* ^*^ = 30°, and (c) *α* ^*^ = 45°. The dashed curves are exponential fittings to the data. The characteristics lengths respectively are 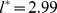 (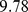), 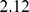 (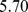), and 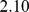 (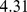) *µm* for directional (re-orientation) flights. A similar qualitative behavior is exhibited by B16F10 cells.(TIFF)

Figure S4 **Turn angle histograms within directional and re-orientation flights performed by Melan A cells.** The turn angle thresholds used were (a) *α* ^*^ = 30°, (b) *α* ^*^ = 45°, and (c) *α* ^*^ = 60°. The same qualitative behavior is exhibited by B16F10 cells.
(TIFF)

Figure S5
**Flight length distributions contaminated for B16F10 cells.** Probability of flights having lengths greater than 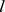 as function of 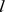 for contaminated B16F10 cells. The turn angle thresholds used were (a) *α* ^*^ = 30°, (b) *α* ^*^ = 45°, and (c) *α* ^*^ = 60°. The dashed curves are exponential fittings to the data. The characteristics length are 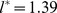 (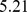), 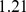 (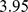), and 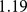 (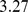) *µm* for directional (re-orientation) flights.(TIFF)

Figure S6 **Turn angle histograms within directional and re-orientation flights performed by contaminated B16F10 cells.** Again, the thresholds used were (a) *α* ^*^ = 30°, (b) *α* ^*^ = 45°, and (c) *α* ^*^ = 60°. The distribution for directional flights is sharper than those for Melan A and B16F10 cells.
(TIFF)

Figure S7
**Turn angle distributions generated by simulated q-Gaussian walks.** The directional flights have characteristic number of steps (a) 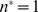 and (b) 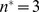. Neatly, the height of the central maximum of 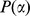 increases with increasing 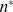.(TIFF)

Figure S8
**Scaling in time of the mean-squared displacement for simulated q-Gaussian walks.** The directional flights have characteristic number of steps (a) 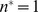 and (b) 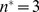. The exponent 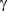 grows faster towards its asymptotic value 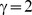 (ballistic diffusion) as 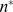 decreases.(TIFF)

Movie S1 **B16F10 dynamics of cell shape alteration and pseudopod protusion.**
(MP4)

Text S1 **Supporting information text file.**
(TEX)
