# Supplementary figures and images for: Normal and Tumoral Melanocytes Exhibit q-Gaussian Random Search Patterns
Source: PLoS One. 2014 Sep 9;9(9):e104253. doi: 10.1371/journal.pone.0104253 (PMC4159146; doi:10.1371/journal.pone.0104253)

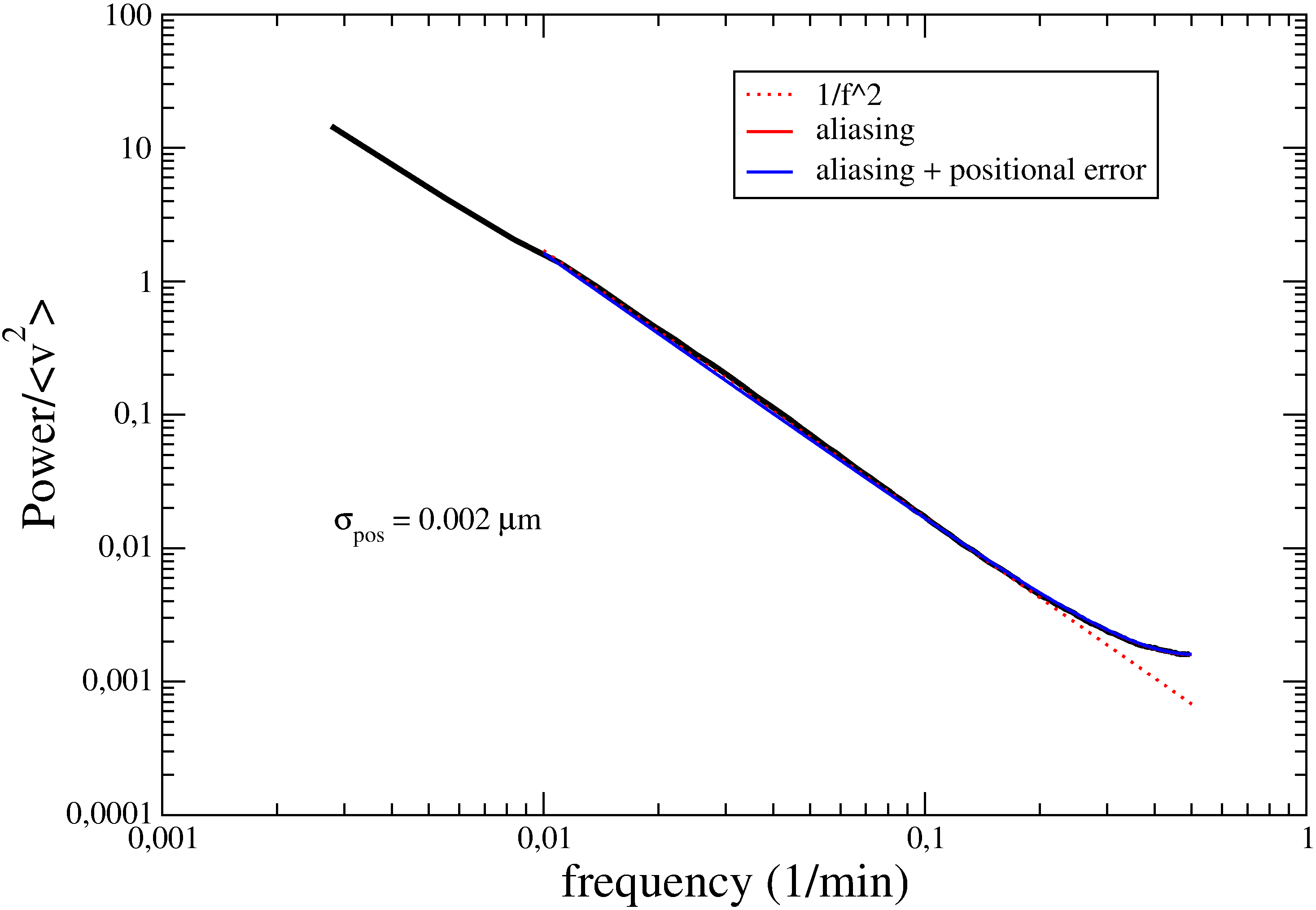

Supplement: Figure S1 — Positional error estimate for B16F10 cells using Li et al aliasing correction. Average spectrum for B16F10 cells (black curve). It was fitted with the aliased spectrum plus the positional noise spectrum (blue curve). The power-law fitting is the dashed red line and the aliased behavior is the red curve. (TIFF) [file pone.0104253.s001.tiff]

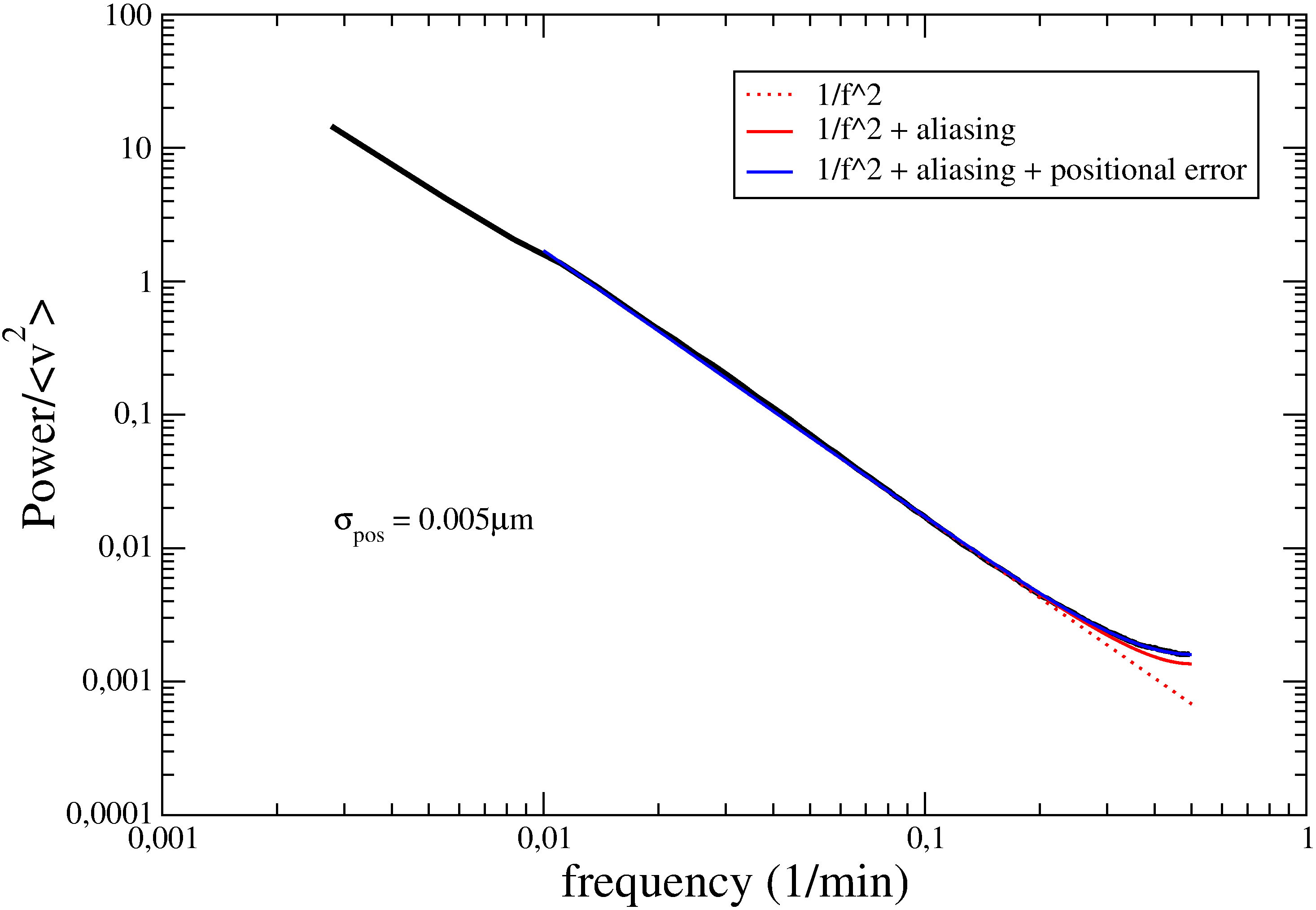

Supplement: Figure S2 — Positional error estimate for B16F10 cells using Kirchner aliasing correction. Average spectrum for B16F10 cells (black curve). It was fitted with the aliased spectrum suggested by Kirchner [34] plus the positional noise spectrum (blue curve). The power-law fitting is the dashed red line and the aliased behavior is the red curve. (TIFF) [file pone.0104253.s002.tiff]

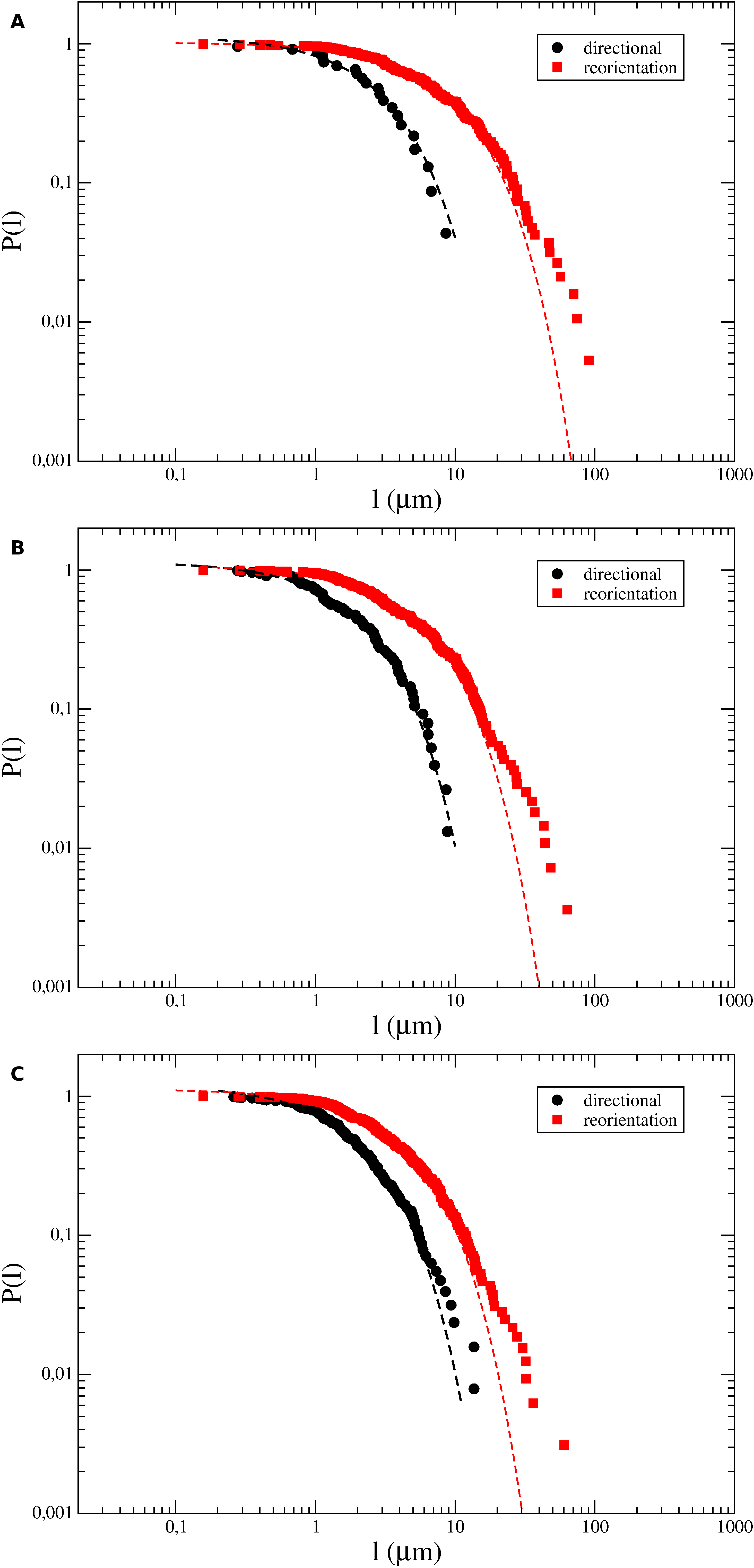

Supplement: Figure S3 — Flight length distributions for Melan A cells. Probability of flights having lengths greater than as function of for Melan A cells. The turn angle thresholds used were (a) α * = 15°, (b) α * = 30°, and (c) α * = 45°. The dashed curves are exponential fittings to the data. The characteristics lengths respectively are (), (), and () µm for directional (re-orientation) flights. A similar qualitative behavior is exhibited by B16F10 cells. (TIFF) [file pone.0104253.s003.tiff]

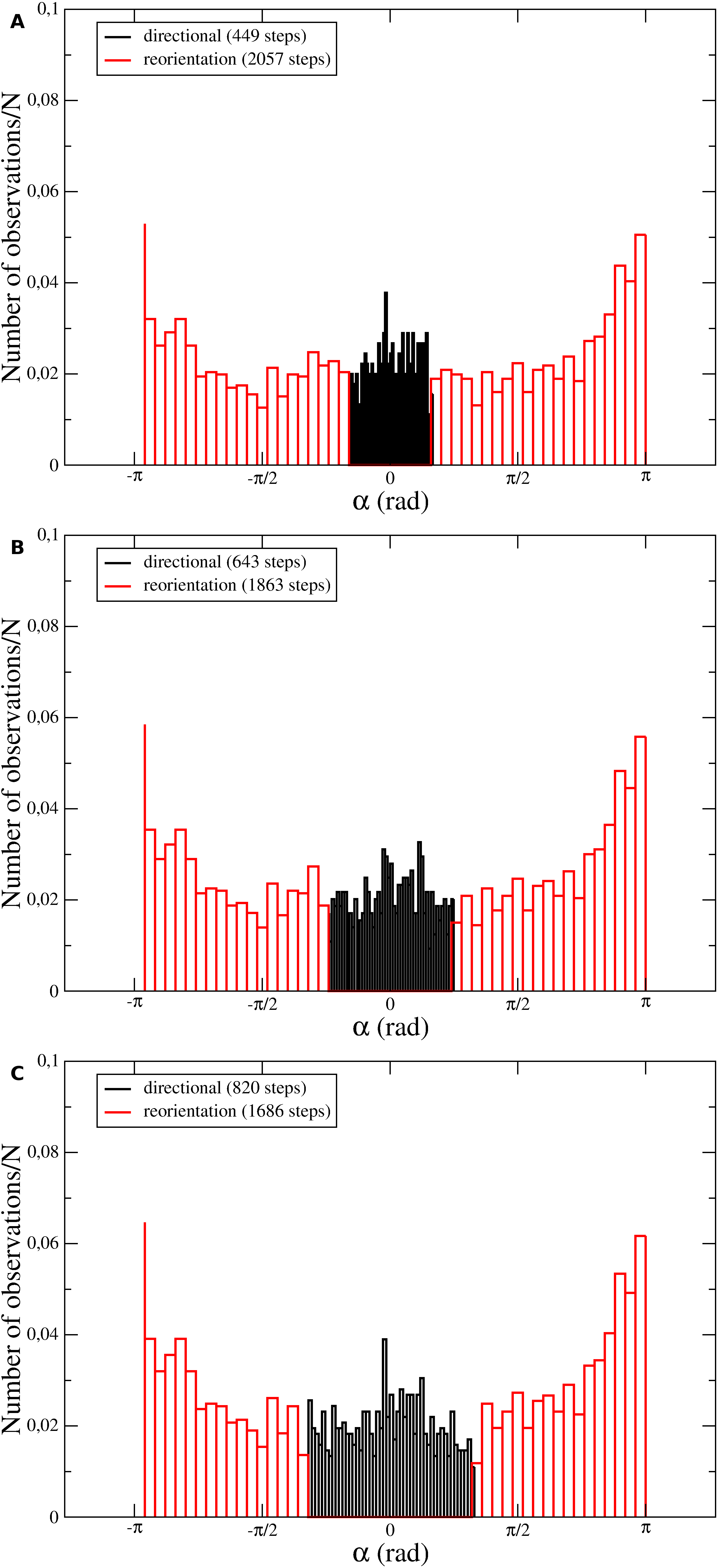

Supplement: Figure S4 — Turn angle histograms within directional and re-orientation flights performed by Melan A cells. The turn angle thresholds used were (a) α * = 30°, (b) α * = 45°, and (c) α * = 60°. The same qualitative behavior is exhibited by B16F10 cells. (TIFF) [file pone.0104253.s004.tiff]

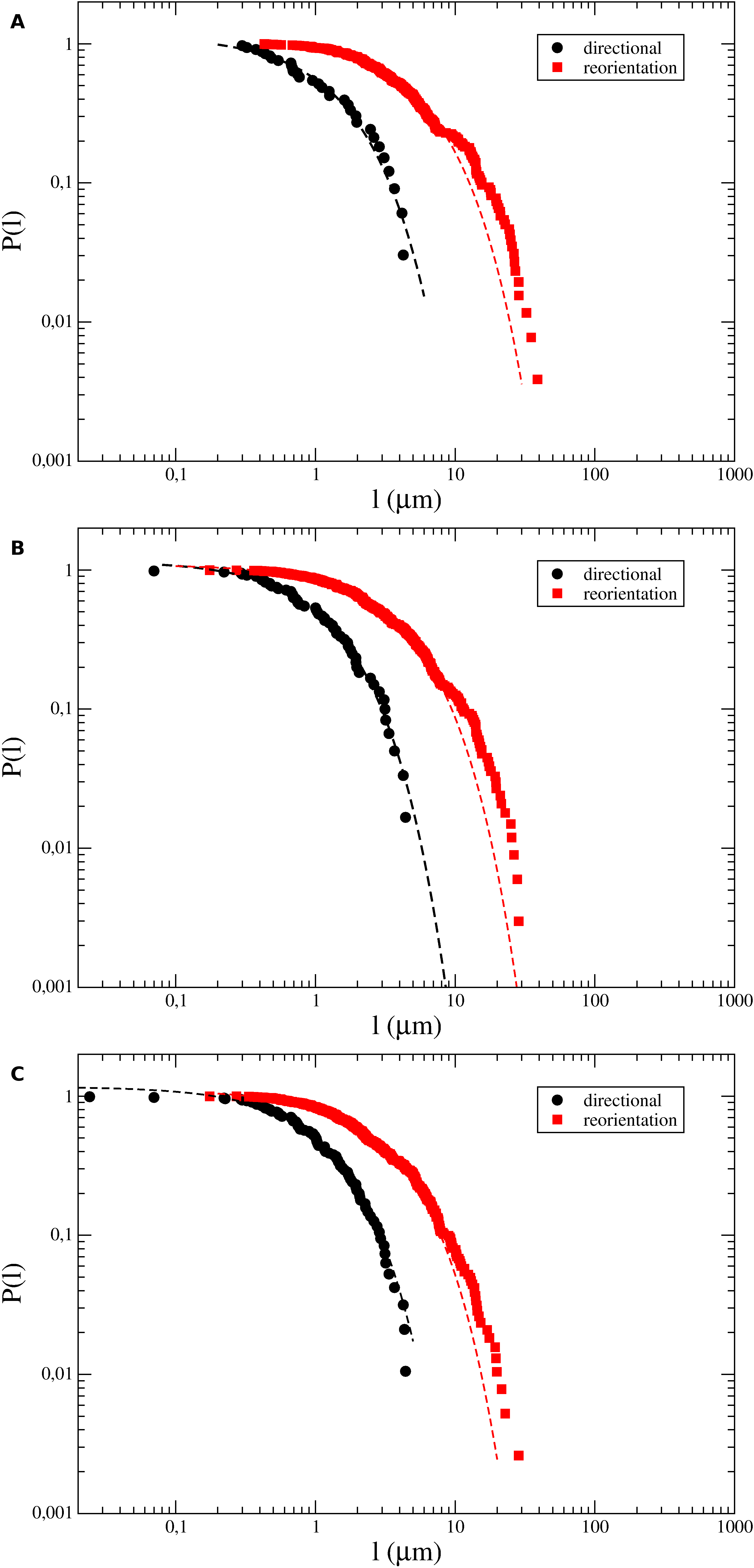

Supplement: Figure S5 — Flight length distributions contaminated for B16F10 cells. Probability of flights having lengths greater than as function of for contaminated B16F10 cells. The turn angle thresholds used were (a) α * = 30°, (b) α * = 45°, and (c) α * = 60°. The dashed curves are exponential fittings to the data. The characteristics length are (), (), and () µm for directional (re-orientation) flights. (TIFF) [file pone.0104253.s005.tiff]

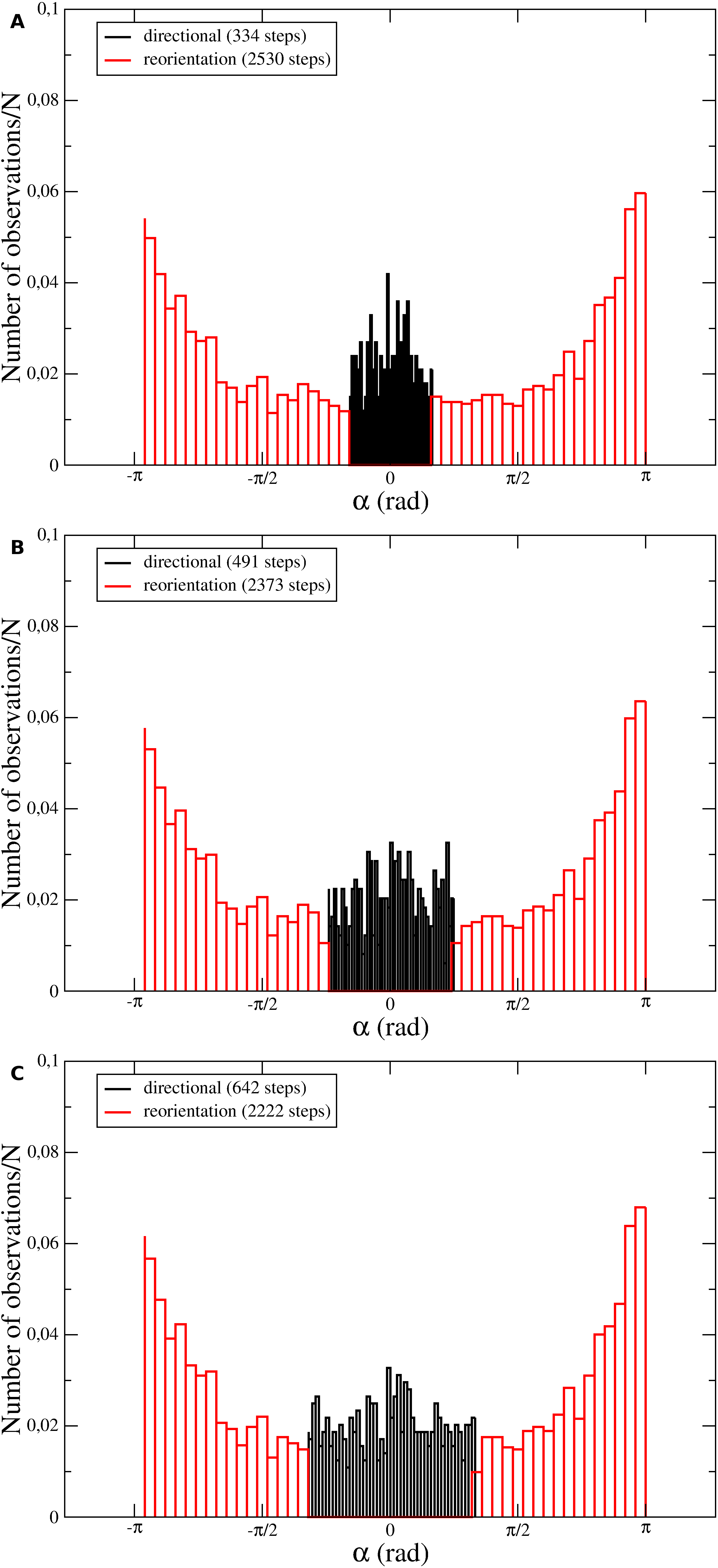

Supplement: Figure S6 — Turn angle histograms within directional and re-orientation flights performed by contaminated B16F10 cells. Again, the thresholds used were (a) α * = 30°, (b) α * = 45°, and (c) α * = 60°. The distribution for directional flights is sharper than those for Melan A and B16F10 cells. (TIFF) [file pone.0104253.s006.tiff]

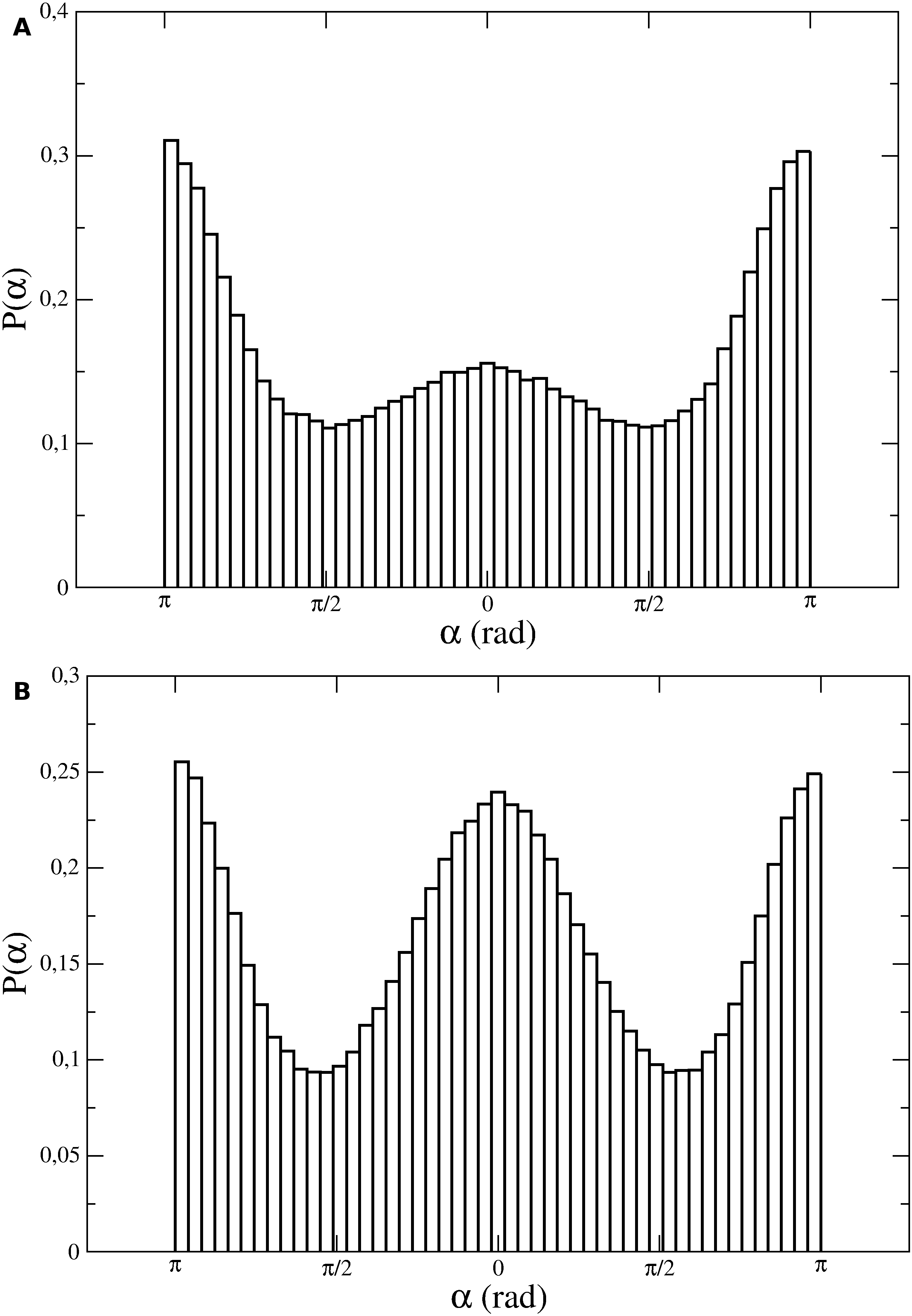

Supplement: Figure S7 — Turn angle distributions generated by simulated q-Gaussian walks. The directional flights have characteristic number of steps (a) and (b) . Neatly, the height of the central maximum of increases with increasing . (TIFF) [file pone.0104253.s007.tiff]

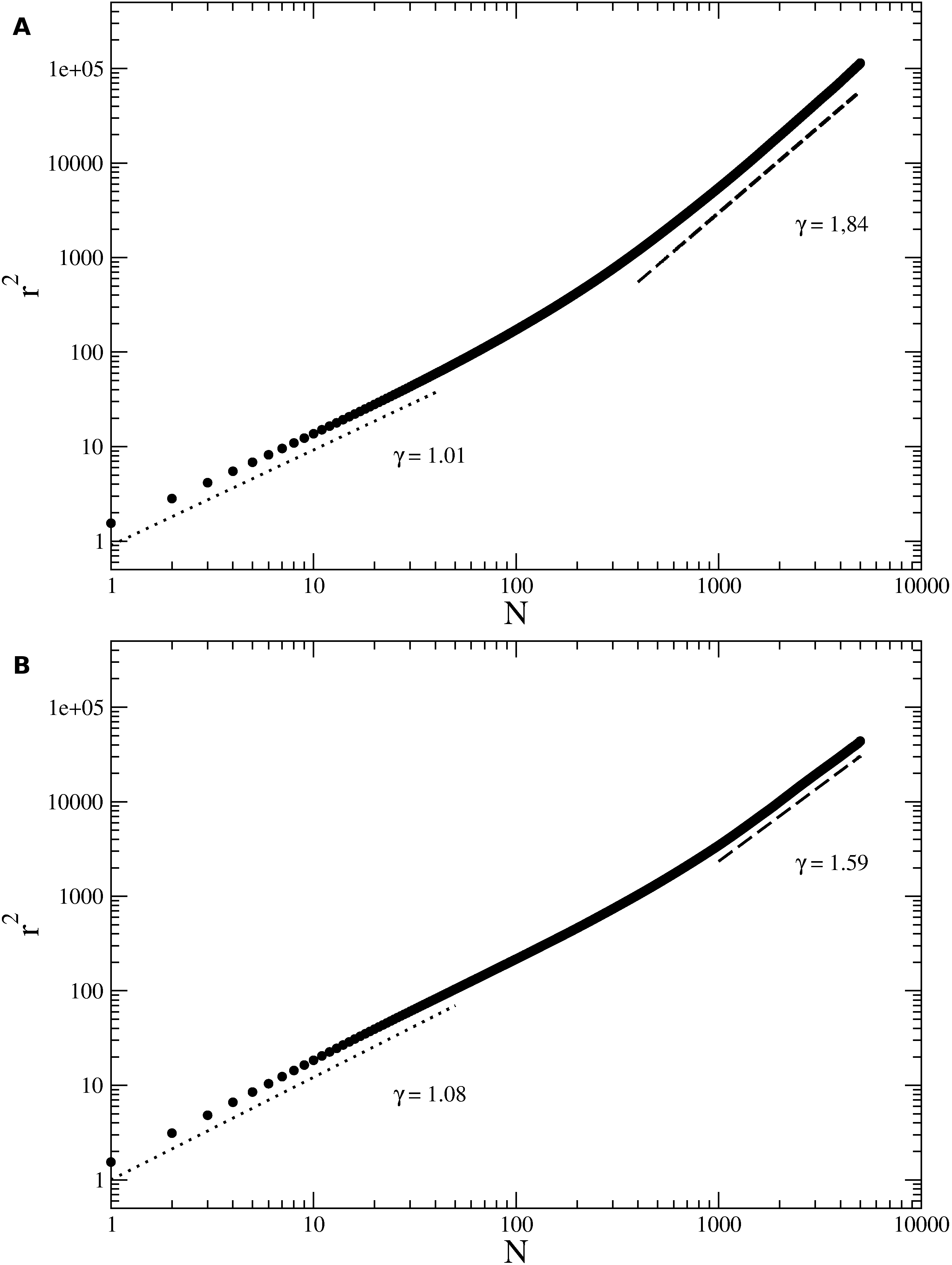

Supplement: Figure S8 — Scaling in time of the mean-squared displacement for simulated q-Gaussian walks. The directional flights have characteristic number of steps (a) and (b) . The exponent grows faster towards its asymptotic value (ballistic diffusion) as decreases. (TIFF) [file pone.0104253.s008.tiff]
